# Saudi Thoracic Society annual award for achievement

**DOI:** 10.4103/1817-1737.49410

**Published:** 2009

**Authors:** Mohamed S. Al-Hajjaj

**Affiliations:** *Professor of Pulmonary Medicine & Consultant Pulmonologist President, Saudi Thoracic Society Respiratory Division, Department of Medicine, King Saud University & King Khalid University Hospital, Riyadh, Saudi Arabia*

Rules and regulations of the Saudi Thoracic Society (STS) have a provision that one of the activities of the society, in order to achieve its objectives, should be to give awards to selected members who fulfill certain requirements. STS administrative committee has agreed to have an annual award to be given to members of the society who make a prominent contribution to the society in any form, whether scientific, organizational or promotional, for the progress of the society.

**The different areas of activities in which the award can be given are as follows:**

**(Category 1) Scientific research**

A scientific research work that is of a measurable extent and judged by the scientific committee of the society as “deserving the STS award.”

Specialties that are to be considered are —

- Pulmonary medicine
- Thoracic surgery
- Pediatric pulmonary diseases
- Critical care medicine
- Sleep disorders medicine
- Respiratory care

**(Category 2) Organizational activity** that is of profound effect on the advancement of the society and judged by the administrative committee as “deserving the STS award.”

Areas to be considered are

- STS conferences, symposia and major scientific activities
- STS-affiliated groups and organizations
- Other administrative activities that are of considerable magnitude

Also included in this category are members who make exceptional contributions in the publication of STS periodicals, website or other promotional activity for furthering the cause of STS and judged by the administrative committee as “deserving the STS award.”

**(Category 3)** Major work and achievement in the area of securing **donations and financial support** for the society from the nonpharmaceutical industry that are of significant help to the society.

## Frequency and time of award distribution

**A total of three (3) awards** are to be handed over to the winners every year during the annual STS conference.

### Nature of the award

The award contains no direct money payment. It will be as follows:

1. A plaque stating the name of the award and the reason for giving it to the winner.
2. A certificate of winning the award, congratulations for the winner and thanking the winner for his contribution to the society.

### Conditions associated with the award

1. The person who can receive the award has to be a member of STS and should have paid the STS annual membership fee.
2. The award is not to be given to a member who has received an award from the society for the same reason.
3. The award is not to be given twice to the same person during the same year, i.e., to a person who is eligible to receive another STS award in the same year.
4. The person who is nominated by the committee to receive the award will be sent a letter informing him/her of the decision of the committee, and he/she has to respond in writing stating that he/she is accepting the award.
5. The person who is to be given the award will be asked to be present during the opening ceremony of the annual STS conference. However, if he/she cannot attend, then he/she has to ask another person to receive it on his/her behalf.

### Procedures for awarding awards

1. A potential winner of the award is to be nominated by —
  - The scientific committee (category 1 only)
  - The establishment where the candidate is working (category 1 only)
  - The administrative committee (categories other than category 1)
2. The scientific research award is to be discussed and approved by the STS scientific committee; the rest of categories are to be discussed and approved by the administrative committee.
3. Voting for the candidate to receive the award by the administrative or scientific committee has to follow the majority rule, ( i.e. more than 50% ) if only one person is nominated. If the number of persons nominated is more than one, then whoever wins more votes is to be considered the winner.
4. All the decisions of the committee have to be finally approved by the chairman of the society to ensure that the procedure is correct and the methodology is intact and according to the STS rules and regulations.
5. STS is to pay for the award plaque, gift and printing of the certificates.
6. Certificates are to be signed by the chairman of the scientific committee and STS chairman (for category 1 awards); and by the chairman and deputy chairman for other categories.

### Cancellation of the award

The award can be cancelled in one or more categories if no nominations are received by the committees at least 2 months before the start of the annual conference, or if the stated committees do not approve the award for the nominations made.

